# Blood Glucose Level, Gestational Diabetes Mellitus and Maternal Birth Season: A Retrospective Cohort Study

**DOI:** 10.3389/fendo.2021.793489

**Published:** 2021-12-16

**Authors:** Dongjian Yang, Jingbo Qiu, An Qin, Lei Chen, Ya Yang, Zhen Huang, Jieyan Qian, Wei Zhu

**Affiliations:** ^1^ International Peace Maternity and Child Health Hospital, School of Medicine, Shanghai Jiao Tong University, Shanghai, China; ^2^ Shanghai Key Laboratory of Embryo Original Diseases, School of Medicine, Shanghai Jiao Tong University, Shanghai, China; ^3^ Department of Infection control, Renji Hospital, School of Medicine, Shanghai Jiao tong University, Shanghai, China

**Keywords:** GDM, birth, blood glucose, EPI - epidemiology, pregnant woman

## Abstract

**Background:**

Previous evidence indicates that birth season is associated with type 2 diabetes in adults. However, information on the association of birth with gestational diabetes mellitus (GDM) is lacking. The present study explores the association between birth seasonality and GDM in East China.

**Methods:**

This retrospective cohort study was conducted at the International Peace Maternal and child health hospital between 2014 and 2019. A total of 79, 292 pregnant women were included in the study after excluding participants with previous GDM, stillbirth, polycystic ovary syndrome, and lack of GDM laboratory records. The multivariate logistic regression model was employed to estimate the odds ratio and 95% confidence interval. After log transformation of blood glucose level, the percentage change and 95% confidence interval were estimated by a multivariate linear model.

**Results:**

The risk of GDM among pregnant women born in spring, autumn, and winter was not significantly different compared to that among participants born in summer. Pregnant women born in autumn had significantly higher 1-hour postprandial blood glucose (PBG-1h) and 2-hour postprandial blood glucose (PBG-2h) levels than pregnant women born in summer. Compared to pregnant women born in August, the PBG-1h level of pregnant women born in October, November, and December increased significantly, whereas the PBG-2h levels of pregnant women born in November and December increased significantly.

**Conclusion:**

Pregnant women born in autumn exhibit higher postprandial blood glucose levels during pregnancy than in those born in summer. The findings provide evidence that exposure to seasonal changes in early life may influence blood glucose metabolism during pregnancy.

## Introduction

Gestational diabetes mellitus (GDM) is a common pregnancy complication. Emerging evidence shows that nearly 1% to 30% of pregnant women experience GDM worldwide (1). The adverse effects of GDM birth outcomes, the health of offspring and mother are documented (2–4). Reducing the incidence of GDM and its impact on health warrants in-depth investigation of underlying mechanisms and associated risk factors.

The impact of prenatal environmental exposure on the risk of type 2 diabetes in adulthood is of increasing concern. Studies have demonstrated that an unfavorable prenatal environment may increase the risk of future health conditions whose outcomes are related to diabetes or GDM (5). Evidence shows that environmental impact during the critical period of prenatal may program the structure and function of organs and tissues critical to glucose homeostasis later in life (6). However, the underlying mechanism of GDM prenatal programming is elusive.

The season or month of birth is a surrogate factor for potential environmental exposure during the perinatal period. Factors associated with seasonal changes include but are not limited to temperature, sunshine, food supply, eating habits, outdoor sports activities, vitamin D synthesis, breastfeeding, infection status, etc (7–13). Although a few studies have investigated the association of birth season or month with adult type 2 diabetes, the results are inconsistent (14–16). Researchers have demonstrated that maternal blood glucose is more sensitive to environmental changes (17). However, data on the relationship between GDM and birth season or month is missing. Here, we retrospectively explored the association of birth season or month of pregnant women with the risk of GDM in Eastern China.

## Methods

### Study Population

The study investigated a retrospective cohort from International Peace Maternity and Child Health Hospital, affiliated with Shanghai Jiaotong University School, from January 2014 to December 2019. Information of 94, 942 medical records from the electronic medical record system, including blood glucose levels, demographic data (date of birth, medical insurance, ethnicity, ward types), disease history, pre-pregnancy BMI, smoking, drinking, birth history (parity, gravidity), etc. were retrieved. The exclusion criteria included pregnant women with no available date of birth, no GDM diagnosis record, previous GDM, multiple births, stillbirths, and abortions. Eventually, 79, 292 pregnant women were investigated (**Figure S1**). Ethical approval was issued by the ethics committee of International Peace Maternity and Child Health Hospital. Written informed consent requirement from patients was waived due to the retrospective design of this study.

### Outcomes

The Oral Glucose Tolerance Test (OGTT) was used to diagnosis GDM at 24-28 weeks of gestation according to the recommendations of the International Association of the Diabetes and Pregnancy Study Groups (IADPSG) (18). Pregnant women took 75 grams of glucose after fasting the night before, and then measured their blood glucose levels at 1 and 2 hours. GDM was defined as fasting blood glucose (FBG) ≥5.1 mmol/L, or/and 1-hour postprandial blood glucose (PBG-1h) ≥10 mmol/L, or/and 2-hour postprandial blood glucose (PBG-2h) ≥8.5 mmol/L.

### Statistical Analysis

The birth season was classified based on astronomical seasons (winter, winter solstice-vernal equinox; spring, spring breeze-summer solstice; summer, summer solstice-autumnal equinox; autumn, autumnal equinox-winter solstice). To assess the effect of seasonal variation in birth on GDM, logistic regression analysis was performed with month and season of birth as the predictor. The reference group was set as the category with the lowest risk of GDM to birth month (FBG: May; PBG-1h and PBG-2h: August) and birth season (Summer). The multivariate logistic regression model of the association between GDM risk and birth season or birth month was adjusted for pregnancy age (years), fetal sex (male or female), the education level (below university, university, or above), drinking (yes, no), smoking (yes, no), family history of diabetes (No, Yes), pre-pregnancy BMI (<18.5, 18.5-23.9, ≥24), parity (1 and ≥2), and gravidity (1, 2, and ≥3), conception method (natural conception, assisted reproduction technology [ART]), medical insurance type (urban or employee, others), ward type (general ward, senior ward), etc.

Logistic regression analysis was conducted after stratification for birth cohort (year of birth ≤ 1985, year of birth>1985), parity (1, ≥2), type of registration (locals, outsiders), and pre-pregnancy BMI (<24, ≥24). Log-transformed blood glucose levels (including PBG-1h, PBG-2h, and FBG) at 24-8 weeks served as the outcome for linear regression analysis in exploring the influence of birth month and birth season on blood glucose. The blood glucose level was closer to a normal distribution after log-transformation (**Figure S2**). Percentage change (PC) and 95% confidence interval (95%CI) represented association.

For sensitivity analysis, the effect of different season classifications (the birth season), including Spring (March, April, May), Summer (June, July, August), and Autumn (September, October, November), Winter (December, January, February), was investigated. To exclude the effect of birth season of offspring on the association between maternal birth season and GDM, we took the pregnancy season as the covariate in the above analyses. The pregnancy season was classified based on astronomical seasons

R software (Version: 3.6.3) was employed for data analysis. Statistical significance was set at two-tailed P<0.05.

## Results

### Participant Characteristics

Of the 79,296 pregnant women were included in this study, the average age of pregnancy was 30.54 ± 3.88 years, the average pre-pregnancy BMI was 20.97 ± 2.75. As shown in **Table 1**, the number of births in the four seasons in descending order is Autumn (29.45%), Summer (25.04%), Winter (24.80%) and Spring (20.72%); the prevalence of GDM in pregnant women born in all seasons is descending from high to low in Spring (14.42%), Autumn (14.36%), Winter (14.34%) and Summer (13.87%); Among all participants, the proportion of primipara was 51.21%, the proportion of drinking during pregnancy was 0.86%, and the proportion of smoking during pregnancy was 0.35%. **Table S1** shows the baseline characteristics of participants by birth season. Pregnant women born in summer exhibited higher pre-pregnancy BMI levels before pregnancy, and higher levels of PBG-1h and PBG-2h in autumn.

**Table 1:** Maternal characteristics of study participants according GDM^a^.

Characteristics	Pregnancy women			P-value
	All	GDM	No-GDM	
Pre-pregnancy BMI, mean (SD)	20.97 (2.75)	22.17 (2.64)	21.14 (3.14)	<0.001^b^
Pregnant age, mean (SD)	30.32 (3.88)	31.86 (3.79)	30.54 (4.09)	<0.001^b^
Ethnicity, n (%)				
han	77994 (98.36)	66901 (85.78)	11093 (14.22)	0.2604
others	1302 (1.64)	1102 (84.64)	200 (15.36)
Fetal sex, n (%)				
Female	38346 (48.36)	32951 (85.93)	5395 (14.07)	0.1824
Male	40950 (51.64)	35052 (85.60)	5898 (14.40)
Gravidity, n (%)				
0	40602 (51.21)	35557 (87.57)	5045 (12.43)	<0.001
1	22418 (28.27)	19007 (84.78)	3411 (15.22)	
>1	16266 (20.52)	13431 (82.57)	2835 (17.43)	
Parity, n (%)				
1	56806 (71.64)	49244 (86.69)	7562 (13.31)	<0.001
>1	22490 (28.36)	18759 (83.41)	3731 (16.59)
Birth Season				
Spring	16427 (20.72)	14059 (85.58)	2368 (14.42)	0.3793
Summer	19852 (25.04)	17099 (86.13)	2753 (13.87)	
Autumn	23352 (29.45)	19999 (85.64)	3353 (14.36)	
Winter	19665 (24.80)	16846 (85.66)	2819 (14.34)	
Ward type, n (%)				
General ward	72398 (91.30)	62111 (85.79)	10287 (14.21)	0.4046
Senior ward	6898 (8.70)	5892 (85.42)	1006 (14.58)
Conception mode, n (%)				
Nature conceived	55462 (69.94)	47947 (86.45)	7515 (13.55)	<0.001
ART	23834 (30.06)	20056 (84.15)	3778 (15.85)
Insurance type, n (%)				
No	62992 (79.46)	54144 (85.95)	8848 (14.05)	0.0021
Yes	16284 (20.54)	13842 (85.00)	2442 (15.00)	
Drinking, n (%)				
No	78611 (99.14)	67417 (85.76)	11194 (14.24)	0.9173
Yes	685 (0.86)	586 (85.55)	99 (14.45)	
Smoking, n (%)				
No	79016 (99.65)	67771 (85.77)	11245 (14.23)	0.1916
Yes	280 (0.35)	232 (82.86)	48 (17.14)	
Family history of diabetes, n (%)				
No	73447 (92.62)	63570 (86.55)	9877 (13.45)	<0.001
Yes	5849 (7.38)	4433 (75.79)	1416 (24.21)
Family history of hypertension, n (%)				
No	64599 (81.47)	55632 (86.12)	8967 (13.88)	<0.001
Yes	14697 (18.53)	12371 (84.17)	2326 (15.83)	
Blood glucose at 24-28 weeks of gestation				
FBG, mean (SD)	4.14 (0.41)	4.53 (0.35)	4.2 (0.57)	<0.001
PBG-1h, mean (SD)	7.57 (1.42)	10.12 (1.06)	7.93 (1.39)	<0.001
PBG-1h, mean (SD)	6.2 (1.42)	8.72 (1.06)	6.56 (1.42)	<0.001

a two independent samples t-test.

b Chi-square test.

### Association of GDM With Birth Months or Seasons

**Figure 1:**
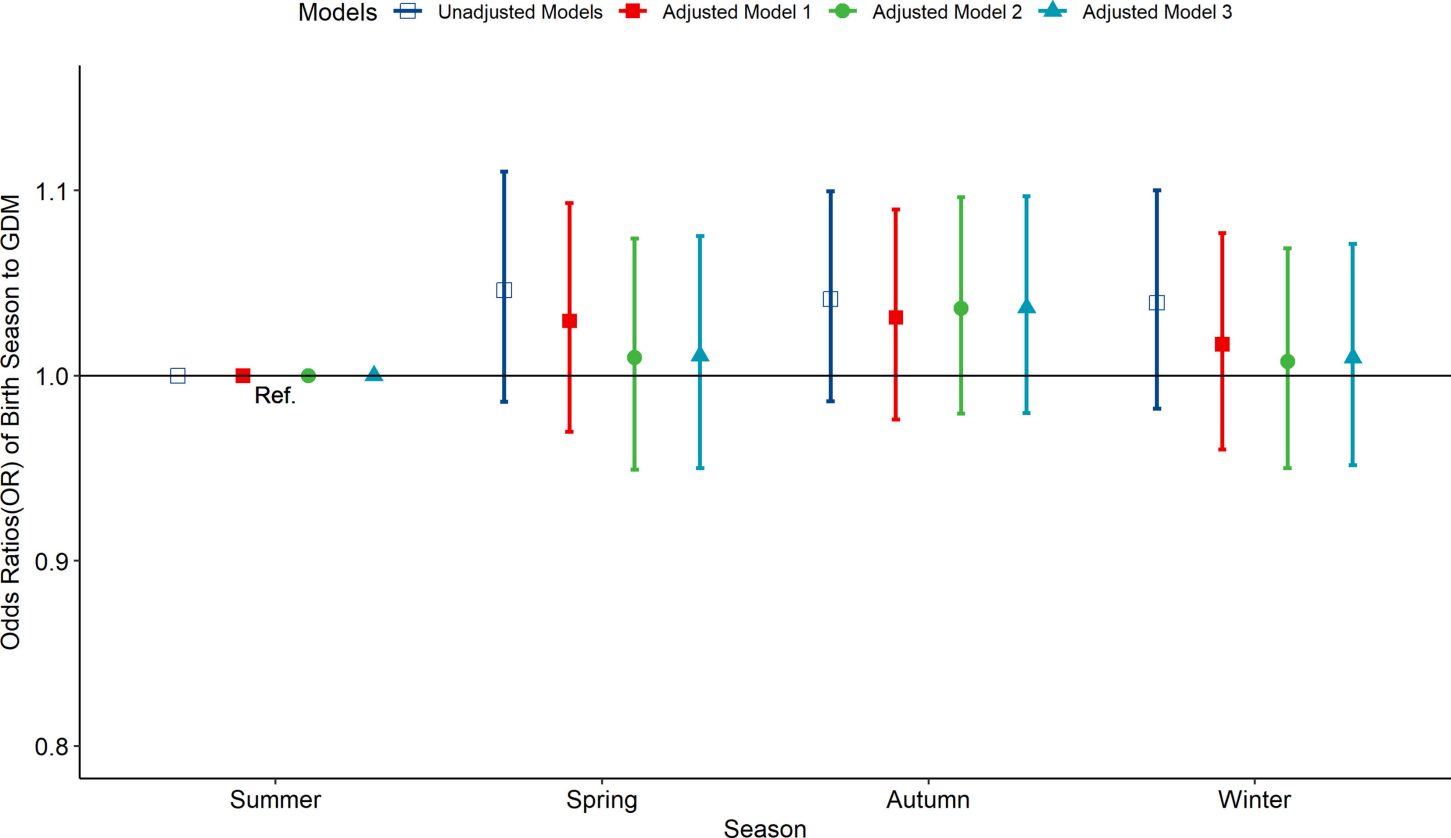
Odds ratio (OR) and 95% confidence interval (95%CI) for GDM of pregnant women according to the season of birth. Adjusted model 1: adjusted for ethnicity, fetal sex, mother education level, ward type, insurance type, pregnant age. Adjusted model 2: in addition to the confounders in adjusted model 1, pre-pregnancy BMI was also adjusted; Adjusted model 3: in addition to the confounders in adjusted mode 2, drinking, smoking, family history of hypertension, family history of diabetes, parity and gravidity were also adjusted. Reference category is born in summer.

**Figure 2:**
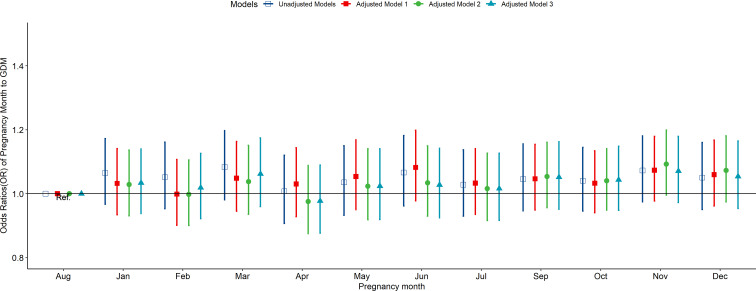
Odds ratio (OR) and 95% confidence interval (95%CI) for GDM of pregnant women according to the month of birth. Adjusted model 1: adjusted for ethnicity, fetal sex, mother education level, ward type, insurance type, pregnant age. Adjusted model 2: in addition to the confounders in adjusted model 1, pre-pregnancy BMI was also adjusted; Adjusted model 3: in addition to the confounders in adjusted mode 2, drinking, smoking, family history of hypertension, family history of diabetes, parity and gravidity were also adjusted. Reference category is born in august.

According to the multivariate-adjusted model, the birth season and month were not significantly associated with the risk factors of GDM (**Figures 1**, **2**). No substantial change in the correlation was reported even after adjusting for pre-pregnancy BMI, family history (hypertension and diabetes), birth history, smoking, and drinking. After further adjusting for pregnancy season, there was no significant correlation between birth season and GDM risk (**Figure S3**). Similarly, when we changed the criteria for the classification of the birth season and conducted another correlation test, results showed no significant association between birth season and GDM risk (**Figure S4**).

### Association of Blood Glucose With Birth Months or Seasons

**Figure 3:**
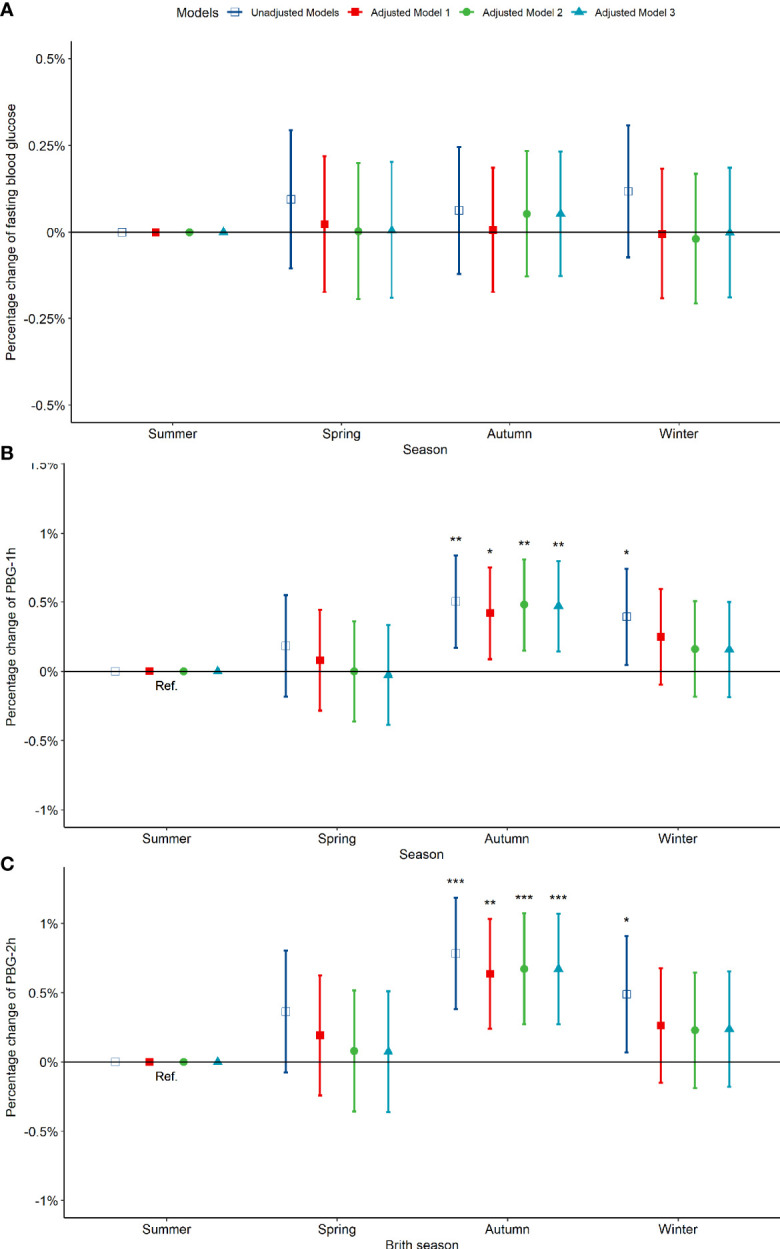
Percentage changes of blood glucose levels [FBG **(A)**, PBG-1h **(B)**, PBG-2h **(C)**] at 24-28 weeks of pregnancy among pregnant women in difference birth month. Adjusted model 1: adjusted for ethnicity, fetal sex, mother education level, ward type, insurance type, pregnant age. Adjusted model 2: in addition to the confounders in adjusted model 1, pre-pregnancy BMI was also adjusted; Adjusted model 3: in addition to the confounders in adjusted mode 2, drinking, smoking, family history of hypertension, family history of diabetes, parity and gravidity were also adjusted. Reference category is born in summer. *p < 0.05; **p < 0.01; ***p < 0.001.

**Figure 4:**
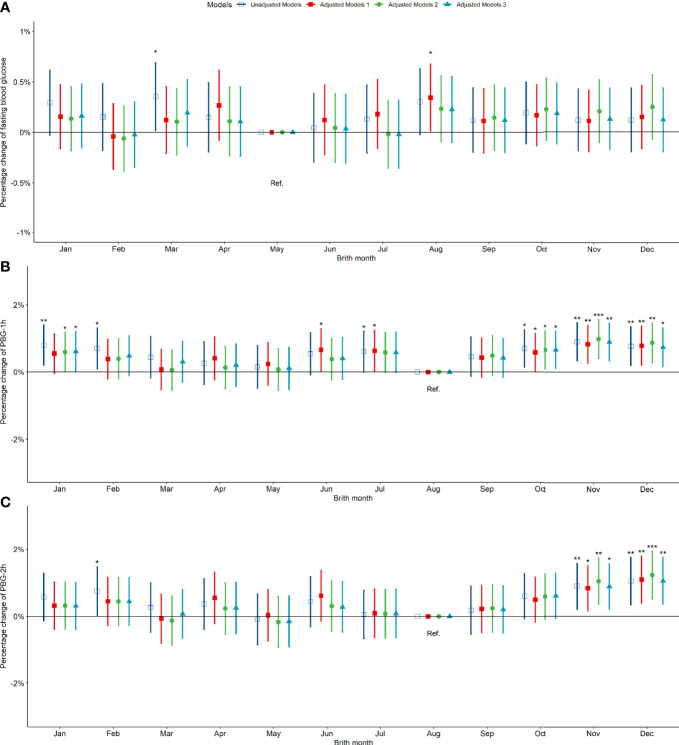
Percentage changes of blood glucose levels [FBG **(A)**, PBG-1h **(B)**, PBG-2h **(C)**] at 24-28 weeks of pregnancy among pregnant women in different birth months. Adjusted model 1: adjusted for ethnicity, fetal sex, mother education level, ward type, insurance type, pregnant age. Adjusted model 2: in addition to the confounders in adjusted model 1, pre-pregnancy BMI was also adjusted; Adjusted model 3: in addition to the confounders in adjusted mode 2, drinking, smoking, family history of hypertension, family history of diabetes, parity and gravidity were also adjusted. Reference category of FBG is born in May; Reference category of PBG-1h and PBG-2h is born in August. *p < 0.05; **p < 0.01; ***p < 0.001.

Compared to pregnant women born in summer, those born in spring, autumn and winter exhibited no significant difference in fasting blood glucose between 24-28 weeks of gestation (**Figure 3A**). However, the PBG-1h level of pregnant women born in the autumn increased significantly by 0.50% (0.17%, 0.84%) and the PBG-2h level increased significantly by 0.78% (0.38%, 1.19%); the PBG-1h and PBG-2h levels of pregnant women born in winter increased significantly; however, the significance disappeared after an adjustment by other factors (**Figures 3B, C**). In addition, the results of the above analysis by seasonal grouping using months show that the results of birth season and blood glucose level are similar to those described above (**Figure S5**). Compared to pregnant women born in August, the PBG-1h levels of pregnant women born in October, November, and December increased significantly by 0.65%, 0.98%, and 0.87%, respectively (**Figure 4B**); the PBG-2h levels of pregnant women born in November, and December increased significantly by 1.05%, and 1.23%, respectively and the correlation existed after multi-factor adjustment (**Figure 4C**); but the FBG level of pregnant women had no significant difference in different birth months (**Figure 4A**).

### Association of GDM With Birth Seasons, Stratified by Birth Cohort

**Figure 5:**
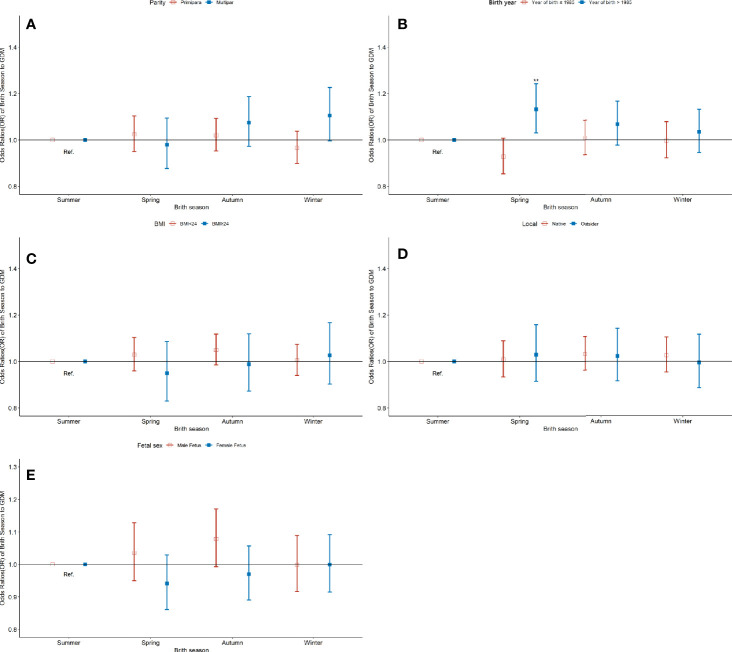
Odds ratio (OR) and 95% confidence interval (95%CI) for GDM of pregnant women according to the season of birth, stratified by parity **(A)**, birth cohort **(B)**, pre-pregnancy BMI **(C)**, type of registration **(D)**, and fetal sex** (E)**. Model adjusted for ethnicity, fetal sex, mother education level, ward type, insurance type, pregnant age, pre-pregnancy BMI, drinking, smoking, family history of hypertension, family history of diabetes, parity and gravidity. Reference category is born in summer. **p < 0.01.

The consistency of the association of birth season with the risk of GDM between different subgroups defined by multiple characteristics of the participants was explored through subgroup analysis. Participants were grouped based on whether they were born locally, parity, age of birth, and pre-pregnancy BMI (**Figures 5A–E**). **Figure 5B** showed that compared to pregnant women born in summer, those born after 1985 exhibited a higher risk of GDM (OR: 1.13, 95% CI: 1.03-1.24). Further analysis of the relationship between birth season and blood sugar level was performed after grouping according to birth year (**Figure S6**). **Figure S6C** demonstrated that for pregnant women born after 1985, those born in autumn and winter have higher PBG-2h levels (PC:1.00%, 95% CI: 0.40%-1.50%).

## Discussion

The relationship between different birth seasons with the risk of GDM during pregnancy has been explored in a large cohort of pregnant women. The hypothesis was that pregnancy at different times of the year and the subsequent changes in the seasonality of various environmental exposure could influence the risk of GDM in the future. The present findings revealed no significant association of the incidence of GDM with the season and month of birth. However, compared with pregnant women born in summer, childbirth in autumn and winter was associated with increased blood glucose in the second trimester. Of note, defined by BMI before pregnancy residence, and parity, this association was consistent across subgroups. Among pregnant women born after 1985, those born in spring showed a higher risk of GDM compared to those born in summer.

To the best of our knowledge, this is the first study to explore the association of birth seasonality with GDM risk during pregnancy. Four studies had previously investigated the association of birth seasonality with the risk of type 2 diabetes in adulthood but the results were inconsistent. A study examining the association of Chinese birth seasonality with the risk of adult type 2 diabetes demonstrated that subjects born in spring, autumn, and winter exhibited a higher risk of diabetes than those born in summer (16). Elsewhere, a study conducted in three regions of Ukraine reported changes in the birth season in 52,214 patients with type 2 diabetes born before 1960, with the peak in April and the lowest in November and December (19). Moreover, in a series of studies conducted by Dutch hospitals on 282 patients with type 2 diabetes aged 30-90 years, when the month of birth was compared to the standard, there were several individuals born with diabetes in the first quarter of this year, and the number of births in the last quarter fell under the birth curve (14). Additional evidence from a prospective study conducted in a cohort of 223,099 adults born in Denmark between 1930 and 1989 demonstrated no association between birth seasonality and the risk of type 2 diabetes (15). The present findings are inconsistent with previous results, which may be ascribed to population specificity of pregnant and lying-in women, GDM diagnosis methods, and differences in regions. In addition, the participants in our study were younger, most of whom had not experienced periods of food scarcity, and younger age is a protective factor for diabetes, which also may account for the inconsistent results.

The above-mentioned studies are aimed at the association between birth season and diabetes in adult. Compelling evidence indicates that, for pregnant women, the blood glucose level increases compensatory during pregnancy to meet the requirement of the developing fetus (20–22). The stability of glucose metabolism is more fragile for an organism exhibiting impaired pancreatic islets and glucose metabolism in early life (23). The present work demonstrated that, compared to pregnant women born in summer, those born in autumn are not associated with a significantly high risk of GDM but their blood glucose levels significantly increased after meals. Studies have shown the association of permanent changes in pancreatic β-cell function or tissue sensitivity to insulin early in life and nutritional changes with insulin resistance and the risk of future diabetes (24, 25). In addition, the effects of pregnancy season on the GDM and blood glucose level, and the effects of subsequent GDM and elevated blood glucose level on offspring glucose metabolism may also be the potential mechanisms. Previous studies (26, 27) have shown the effect of pregnancy season on GDM. Furthermore, seasonal changes in food types, food nutritional value, and seasonal changes in eating habits may increase the risk of type 2 diabetes in adulthood in food-deficit areas or years (16, 28–30). However, herein, pregnant women were, in most cases, born in areas with better economic development after 1975. Although the changes in early life glucose metabolism caused by nutritional deficiencies are not so obvious, they are likely to influence postprandial blood glucose levels because they impact insulin sensitivity. In addition, the year of birth of pregnant women in this study includes the era of China’s rapid economic development, and the early nutritional supply of pregnant women born in different years is also very different. As such, further investigation is warranted to explore the underlying mechanism of association between birth season and GDM.

An interesting finding of this work is that after birth year stratification, pregnant women born in spring after 1985 have a higher risk of GDM than those born in summer. After 1985, Eastern China was experiencing rapid economic development (31, 32), therefore, issues with food shortage were rare. Compared to pregnant women born in summer, those born in spring experienced cold weather during the late pregnancy and newborn period and were exposed to environmental pollution during the Chinese Spring Festival (33–35). These events may also have impacted glucose metabolism function in their early life (36, 37), but these conclusions need further exploration.

Although this research conducted in East China provides some intriguing findings, some limitations must be acknowledged. First, we did not explore factors, including the lifestyle of the participants, and therefore could not correct for the impact of lifestyle. Second, the study lacks information on exposure and material characteristics at birth; such as birth weight, maternal nutrition during pregnancy, parental socioeconomic status, and breastfeeding. Third, the division of birth season is based on the date of birth, and some information may be inaccurate. For instance, a few people in the analyzed regions habitually use the date of the lunar calendar as their date of birth. Lastly, a proportion of the explored population is small which may influence participants’ birth season classification. Also, subjects who had previously suffered from GDM were excluded as we could not obtain information on previous GDM. This may underestimate the effect of birth season on GDM, except that the part of the excluded population is less than 1% of the total population.

## Conclusion

This large retrospective cohort study demonstrates that compared to pregnant women born in summer, those born in autumn and winter have significantly higher blood glucose levels at 1 hour and 2 hours after a meal in the second trimester. The findings provide strong evidence that exposure to some degree of seasonal changes early in life potentially impacts glucose metabolism during pregnancy. However, a further in-depth research is warranted to verify our findings and clarify the underlying mechanism.

## Data Availability Statement

The raw data supporting the conclusions of this article will be made available by the authors, without undue reservation.

## Author Contributions

DY and YY conducted the analyses. DY and AQ wrote the manuscript. WZ, LC, and ZH contributed to data collection. DY, JBQ, and WZ contributed to study design. WZ and DY edited the manuscript. All authors contributed to the article and approved the submitted version.

## Funding

This work was funded by the Shanghai Municipal Education Commission-Gaoyuan Nursing Grant Support (Hlgy1803sjk); National Key Research and Development Program of China (2019YFC1005106); Appropriate technology project of International Peace Maternity and Child Health Hospital of China Welfare Institute (CR2018SY02).

## Conflict of Interest

The authors declare that the research was conducted in the absence of any commercial or financial relationships that could be construed as a potential conflict of interest.

## Publisher’s Note

All claims expressed in this article are solely those of the authors and do not necessarily represent those of their affiliated organizations, or those of the publisher, the editors and the reviewers. Any product that may be evaluated in this article, or claim that may be made by its manufacturer, is not guaranteed or endorsed by the publisher.
