# Supplementary material for: Blood Glucose Level, Gestational Diabetes Mellitus and Maternal Birth Season: A Retrospective Cohort Study
Source: Front Endocrinol (Lausanne). 2021 Dec 16;12:793489. doi: 10.3389/fendo.2021.793489 (PMC8716549; doi:10.3389/fendo.2021.793489)
Supplement: Supplementary file 1 [file DataSheet_1.docx]

Table S1. Maternal characteristics of study participants according seasons

| Characteristics | Pregnancy women | | | | |
| --- | --- | --- | --- | --- | --- |
|  | All | Spring | Summer | Autumn | Winter |
| Pre-pregnancy BMI, mean(SD) | 21.14(2.75) | 21.13(2.79) | 21.21(2.79) | 21.10(2.7) | 21.14(2.75) |
| Pregnant age, mean(SD) | 30.54(3.88) | 30.39(3.86) | 30.59(3.93) | 30.53(3.84) | 30.66(3.9) |
| Ethnicity, n(%) |  |  |  |  |  |
| han | 77994(98.36) | 19515(98.3) | 16116(98.11) | 23028(98.61) | 19335(98.32) |
| others | 1302(1.64) | 337(1.7) | 311(1.89) | 324(1.39) | 330(1.68) |
| Fetal sex, n(%) |  |  |  |  |  |
| Female | 38346(48.36) | 9610(48.41) | 7987(48.62) | 11253(48.19) | 9496(48.29) |
| Male | 40950(51.64) | 10242(51.59) | 8440(51.38) | 12099(51.81) | 10169(51.71) |
| Gravidity, n(%) |  |  |  |  |  |
| 0 | 40602(51.21) | 10196(51.36) | 8399(51.14) | 11838(50.7) | 10169(51.72) |
| 1 | 22418(28.27) | 5605(28.24) | 4673(28.45) | 6649(28.48) | 5491(27.93) |
| >1 | 16266(20.52) | 4050(20.4) | 3352(20.41) | 4863(20.83) | 4001(20.35) |
| Parity, n(%) |  |  |  |  |  |
| 1 | 56806(71.64) | 14305(72.06) | 11782(71.72) | 16554(70.89) | 14165(72.03) |
| >1 | 22490(28.36) | 5547(27.94) | 4645(28.28) | 6798(29.11) | 5500(27.97) |
| Ward type, n(%) |  |  |  |  |  |
| General ward | 72398(91.3) | 18096(91.15) | 14987(91.23) | 21306(91.24) | 18009(91.58) |
| Senior ward | 6898(8.7) | 1756(8.85) | 1440(8.77) | 2046(8.76) | 1656(8.42) |
| Conception mode, n(%) | |  |  |  |  |
| Nature conceived | 55462(69.94) | 13851(69.77) | 11534(70.21) | 16333(69.94) | 13744(69.89) |
| ART | 23834(30.06) | 6001(30.23) | 4893(29.79) | 7019(30.06) | 5921(30.11) |
| Insurance type, n(%) |  |  |  |  |  |
| No | 62992(79.46) | 15776(79.5) | 12948(78.83) | 18633(79.82) | 15635(79.51) |
| Yes | 16284(20.54) | 4068(20.5) | 3477(21.17) | 4711(20.18) | 4028(20.49) |
| Drinking, n(%) |  |  |  |  |  |
| No | 78611(99.14) | 19670(99.08) | 16278(99.09) | 23159(99.17) | 19504(99.18) |
| Yes | 685(0.86) | 182(0.92) | 149(0.91) | 193(0.83) | 161(0.82) |
| Smoking, n(%) |  |  |  |  |  |
| No | 79016(99.65) | 19775(99.61) | 16372(99.67) | 23274(99.67) | 19595(99.64) |
| Yes | 280(0.35) | 77(0.39) | 55(0.33) | 78(0.33) | 70(0.36) |
| Family history of diabetes, n(%) | |  |  |  |  |
| No | 73447(92.62) | 18391(92.64) | 15201(92.54) | 21616(92.57) | 18239(92.75) |
| Yes | 5849(7.38) | 1461(7.36) | 1226(7.46) | 1736(7.43) | 1426(7.25) |
| Family history of hypertension, n(%) | | |  |  |  |
| No | 64599(81.47) | 16250(81.86) | 13323(81.1) | 18982(81.29) | 16044(81.59) |
| Yes | 14697(18.53) | 3602(18.14) | 3104(18.9) | 4370(18.71) | 3621(18.41) |
| GDM diagnosis |  |  |  |  |  |
| GDM | 11293(14.24) | 2368(14.42) | 2753(13.87) | 3353(14.36) | 2819(14.34) |
| No-GDM | 68003(85.76) | 14059(85.58) | 17099(86.13) | 19999(85.64) | 16846(85.66) |
| Blood glucose at 24-28 weeks of gestation | | | |  |  |
| FBG, mean(SD) | 4.2(0.41) | 4.2(0.41) | 4.2(0.42) | 4.2(0.41) | 4.2(0.42) |
| 1h-PBG, mean(SD) | 7.93(1.42) | 7.91(1.42) | 7.93(1.43) | 7.95(1.42) | 7.94(1.44) |
| 2h-PBG, mean(SD) | 6.56(1.42) | 6.54(1.42) | 6.56(1.42) | 6.58(1.41) | 6.57(1.42) |


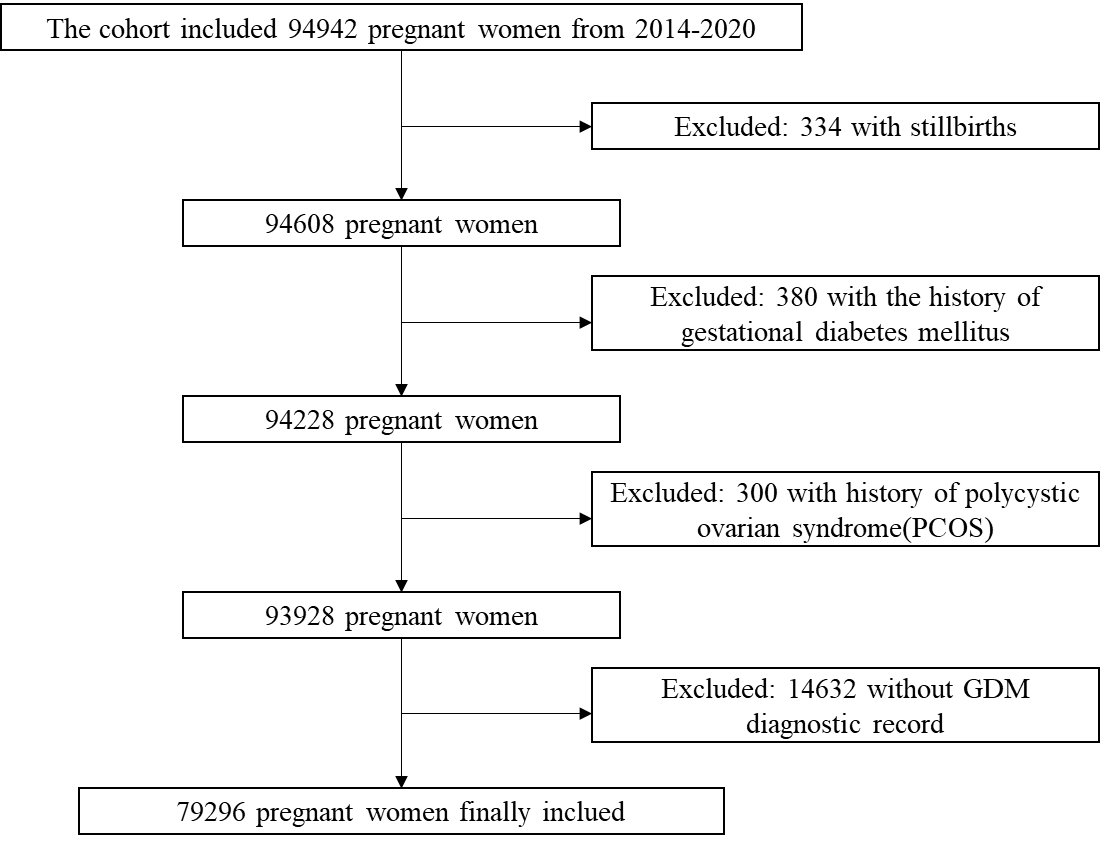


Figure S1. Study flow of the study population.


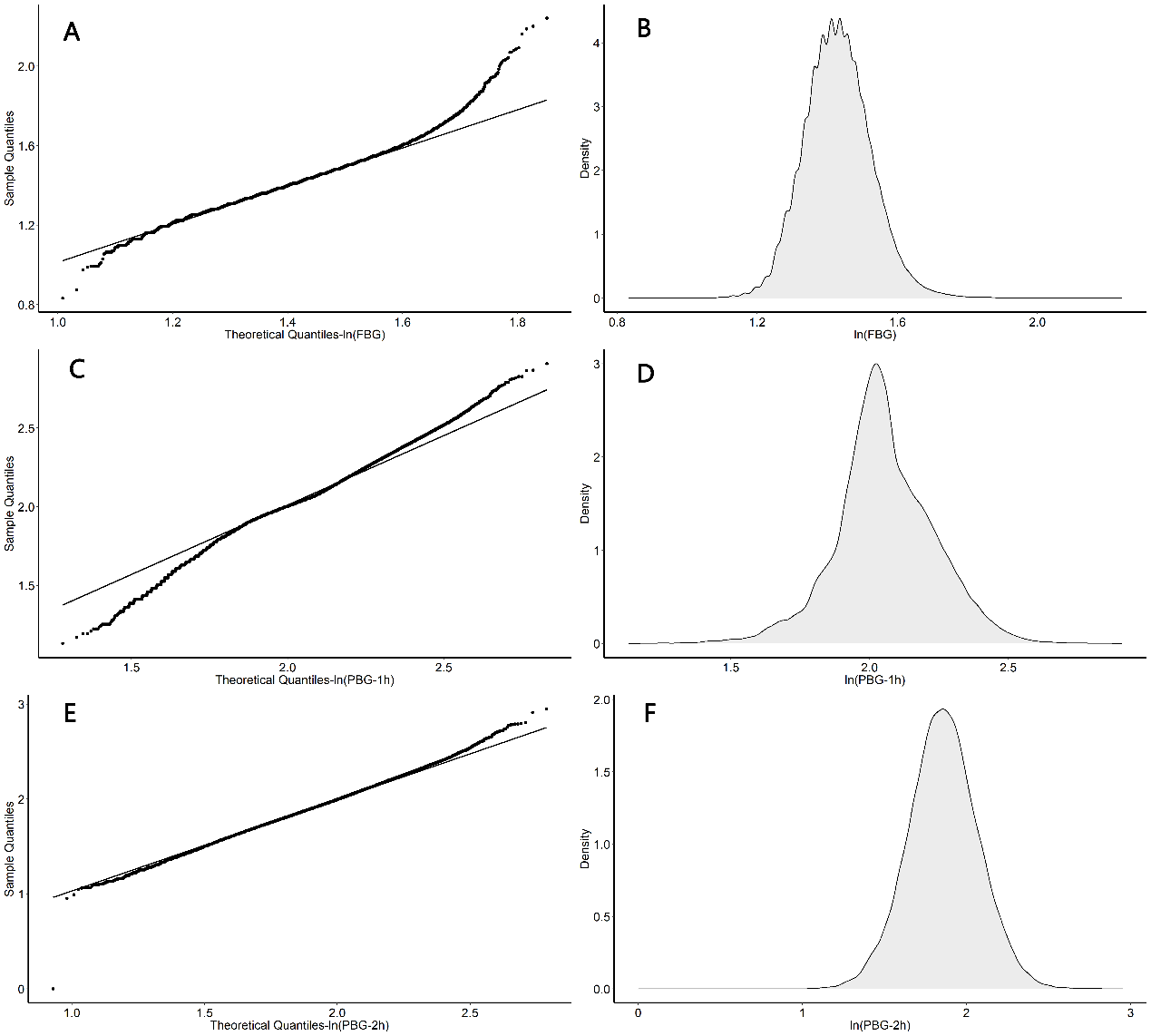


Figure S2 The Q-Q plots and density plots of blood glucose levels[FBG (A and B), PBG-1h (C and D), PBG-2h (E and F)] after log-transformation at 24-28 weeks of pregnancy among pregnant women


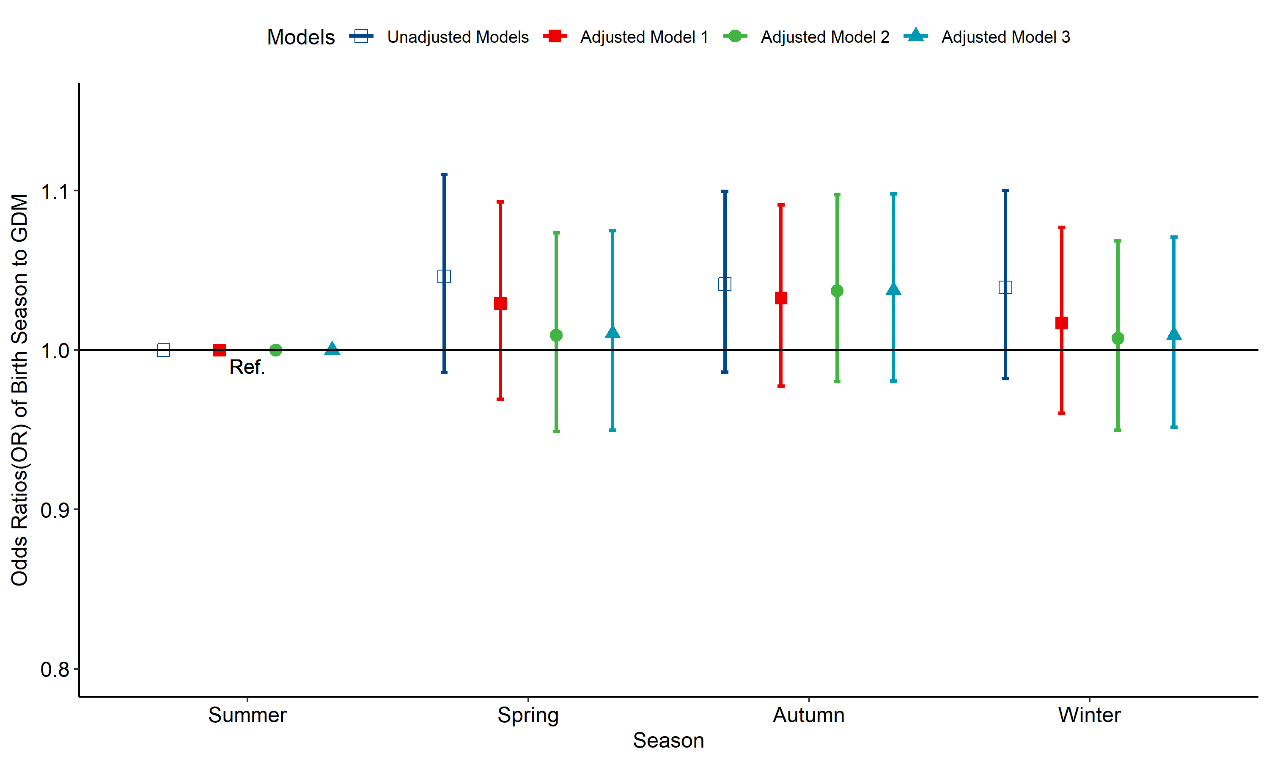


Figure S3 Odds ratio (OR) and 95% confidence interval (95%CI) for GDM of pregnant women according to the season of birth, adjusted by pregnancy season. Adjusted model 1: adjusted for ethnicity, fetal sex, mother education level, ward type, insurance type, pregnancy age and pregnancy season. Adjusted model 2: in addition to the confounders in adjusted model 1, pre-pregnancy BMI was also adjusted; Adjusted model 3: in addition to the confounders in adjusted mode 2, drinking, smoking, family history of hypertension, family history of diabetes, parity and gravidity were also adjusted. Reference category is born in summer.


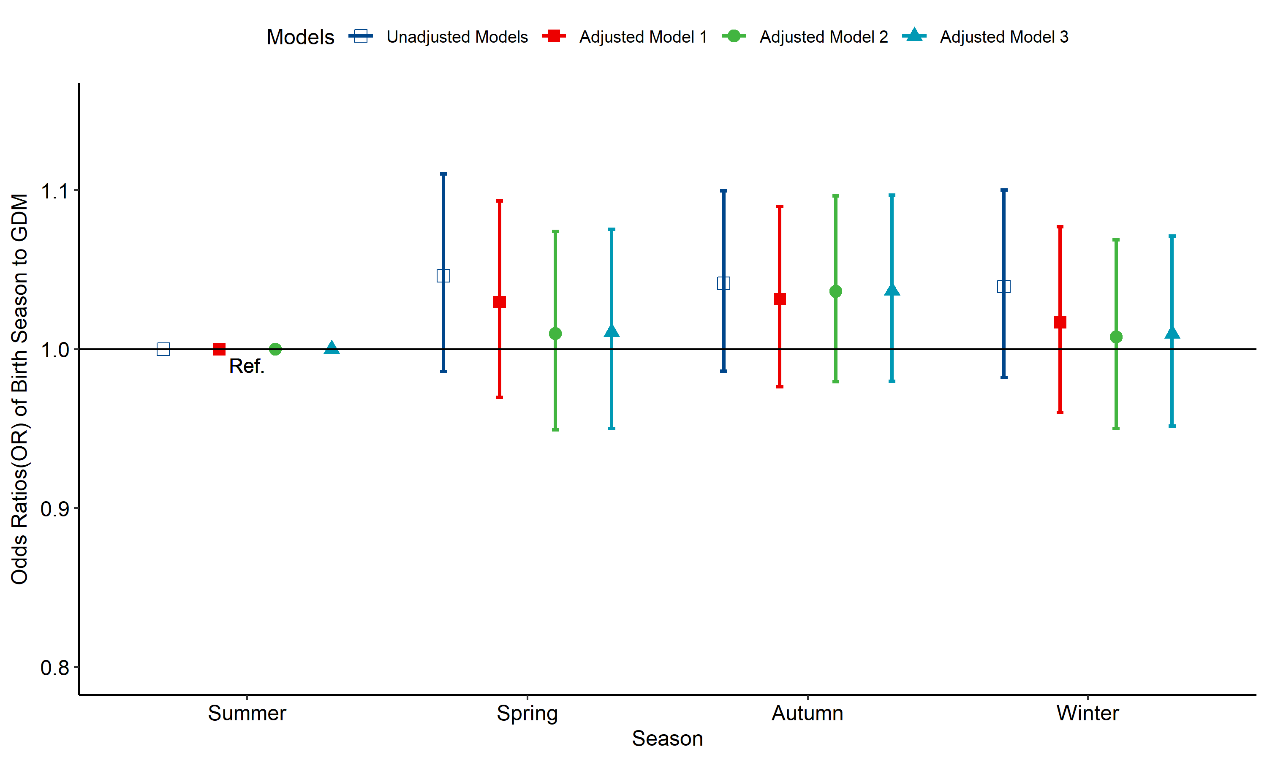


Figure S4 Odds ratio (OR) and 95% confidence interval (95%CI) for GDM of pregnant women according to the season of birth (classification by month). Adjusted model 1: adjusted for ethnicity, fetal sex, mother education level, ward type, insurance type, pregnant age. Adjusted model 2: in addition to the confounders in adjusted model 1, pre-pregnancy BMI was also adjusted; Adjusted model 3: in addition to the confounders in adjusted mode 2, drinking, smoking, family history of hypertension, family history of diabetes, parity and gravidity were also adjusted. Reference category is born in summer.


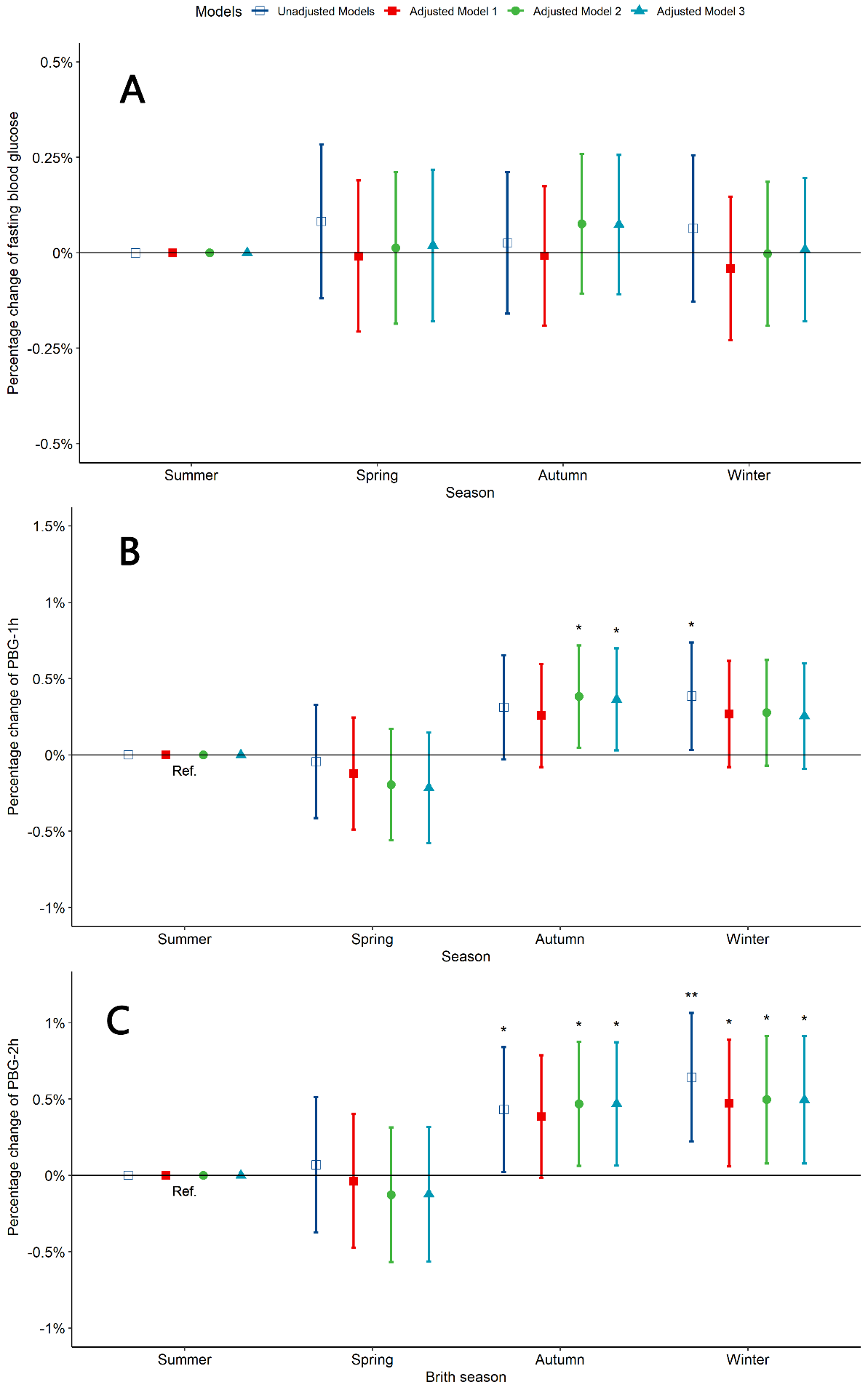


Figure S5 Percentage changes of blood glucose among pregnant women in different birth season (classification by month). Adjusted model 1: adjusted for ethnicity, fetal sex, mother education level, ward type, insurance type, pregnant age. Adjusted model 2: in addition to the confounders in adjusted model 1, pre-pregnancy BMI was also adjusted; Adjusted model 3: in addition to the confounders in adjusted mode 2, drinking, smoking, family history of hypertension, family history of diabetes, parity and gravidity were also adjusted. Reference category is born in summer.


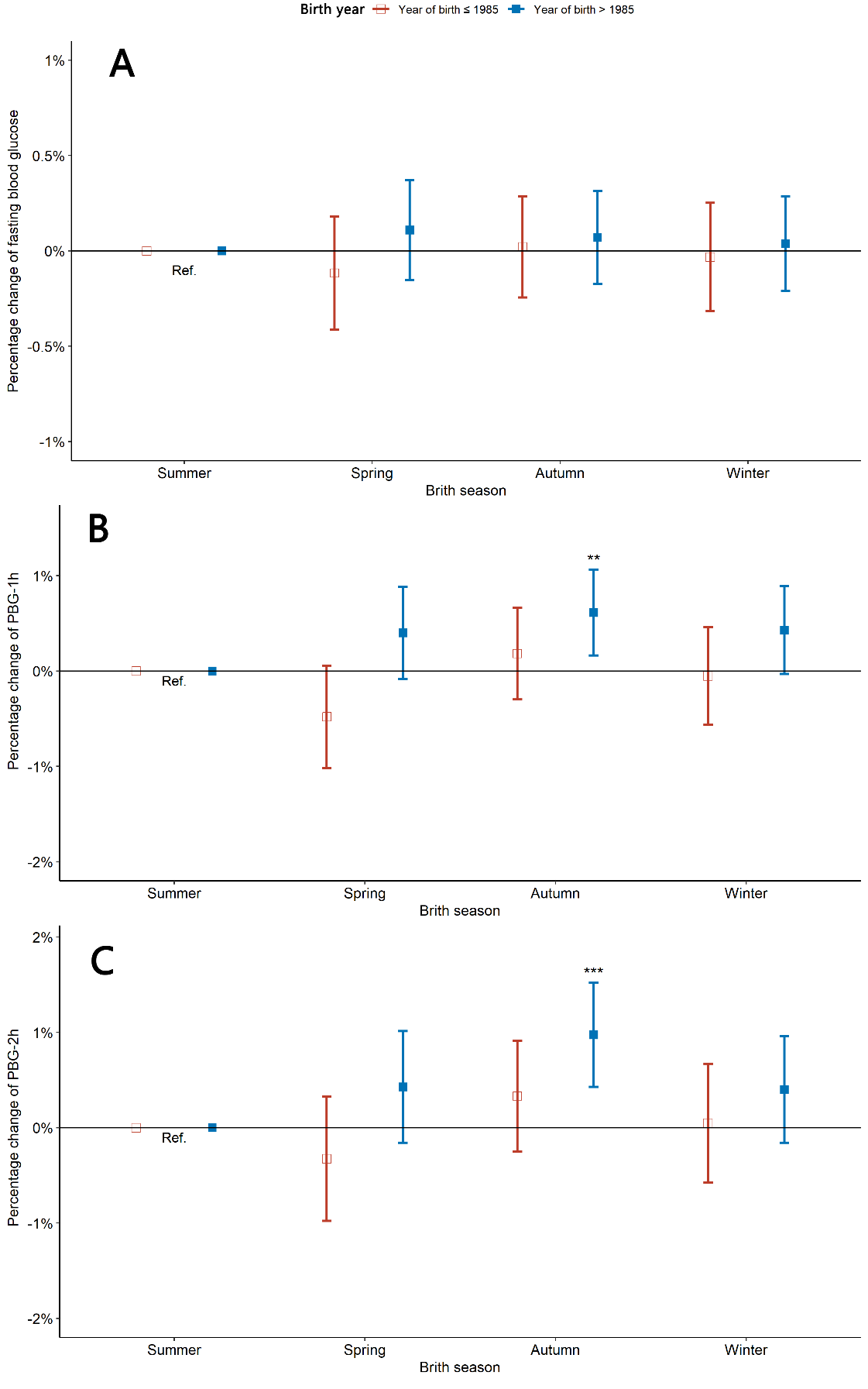


Figure S6 Percentage changes of blood glucose in pregnant women in different birth months, stratified by birth cohort. Model adjusted for ethnicity, fetal sex, mother education level, ward type, insurance type, pregnant age, pre-pregnancy BMI, drinking, smoking, family history of hypertension, family history of diabetes, parity and gravidity. Reference category is born in summer.
